# Determinants of growth differentiation factor 15 in patients with stable and acute coronary artery disease. A prospective observational study

**DOI:** 10.1186/s12933-016-0375-8

**Published:** 2016-04-08

**Authors:** Serdar Farhan, Matthias K. Freynhofer, Ivan Brozovic, Veronika Bruno, Birgit Vogel, Ioannis Tentzeris, Sabina Baumgartner-Parzer, Kurt Huber, Alexandra Kautzky-Willer

**Affiliations:** 3rd Department of Medicine, Cardiology, Wilhelminen Hospital, Montleartstrasse 37, A-1160 Vienna, Austria; Department of Obstetrics and Gynecology, Wilhelminen Hospital, Vienna, Austria; 3rd Department of Medicine, Division of Endocrinology and Metabolism, Medical University of Vienna, Vienna, Austria

**Keywords:** Growth differentiation factor 15, Acute coronary syndrome, Coronary artery disease, Diabetes mellitus, Acute hyperglycemia

## Abstract

**Background:**

Growth differentiation factor 15 (GDF-15) is a member of the transforming growth factor ß family and has been associated with inflammation, cancer, aging, diabetes mellitus (DM) and atherosclerosis. Determinants of GDF-15 have been investigated in several conditions. We aimed to investigate determinants of GDF-15 plasma levels in patients with angiographically proven coronary artery disease (CAD).

**Methods:**

Four hundred and seventy three consecutive patients with CAD were investigated between May 2009 and February 2011. Patients were separated into those with stable CAD (SCAD) and with ST-elevation and non-ST-elevation myocardial infarction (STEMI and NSTEMI). Blood samples for determination of GDF-15 were obtained before coronary angiography. Determinant of GDF-15 levels were analyzed by logistic regression analysis in unadjusted and adjusted models. Study endpoints were cardiovascular death (CV-death), myocardial infarction, unstable angina, unplanned revascularization, stent thrombosis and stroke assessed at a mean follow-up of 188 (177.2–243) days.

**Results:**

Overall median and (25–27th percentile) GDF-15 level was 1212.8 pg/ml (833.2–1957 pg/ml). GDF-15 was significantly higher in STEMI compared to SCAD and NSTEMI groups (P < 0.0001). In a multivariate regression analysis advanced age, DM, acute hyperglycemia (AHG), CRP and chronic kidney disease (CKD) were independent predictors of elevated GDF-15 levels (P < 0.05). Receiver operating curve analysis of GDF-15 for prediction of CV-death showed an area under the curve of 0.852 with a confidence interval of 0.745-0.960, P < 0.0001. The estimated cut-off was 2094.6 pg/ml with a sensitivity of 76 % and specificity of 80 %.

**Conclusion:**

In patients with CAD undergoing PCI with stent implantation, GDF-15 is determined by advanced age, acute and chronic hyperglycemia, inflammation and CKD. GDF-15 is a valuable predictor of CV-death in a population of CAD patients after PCI.

## Background

Growth differentiation factor 15 (GDF-15) is a member of the transforming growth factor family [1]. GDF-15 has been described in various conditions [2–4]. GDF-15 has been introduced as a prognostic marker for patients with coronary artery disease (CAD) especially in the case of acute non-ST-elevation myocardial infarction (NSTEMI) [5–7]. Additionally GDF-15 has been linked to hyperglycemia and diabetes mellitus (DM) [4, 8]. GDF-15 was investigated also as a predictor of future insulin resistance and impaired glucose control in obese non-diabetic subjects [9]. Furthermore, GDF-15 was linked to impaired fasting glucose [8]. Additionally GDF-15 predicted worsening albuminuria in patients with DM [3].

Few data are available on determinants of GDF-15 in patients with CAD. Therefore we aimed to investigate GDF-15 in patients with CAD undergoing percutaneous coronary intervention (PCI) with stent implantation.

## Methods

In total 473 consecutive patients from the WIlhelminen hospital Monitoring of Antiplatelet Activity (WILMAA)-registry between May 2009 and February 2011 were included in this prospective observational investigation [10]. Briefly, consecutive patients, with percutanous coronary intervention (PCI) and coronary stenting due to stable coronary artery disease (SCAD) with positive ischemia testing (treadmill examination, dobutamine stress echocardiography or single-photon-emission computed tomography) or acute coronary syndrome (ACS), aged >18 years were eligible for inclusion in the present study. ACS was diagnosed according to the guidelines of the European Society of Cardiology (ESC) [11, 12]. Implantation of drug eluting or bare metal stents, and the use of accompanying antithrombotic regimens were based on the decision of the treating interventionist. The city of Vienna ethic committee approved the study and all patients gave their informed consent.

Patients were divided according their presentation into those with SCAD as well as ST-elevation myocardial infarction (STEMI) or NSTEMI.

*Definitions* Acute hyperglycemia (AHG) was defined as glucose concentration of ≥140 mg/dl in absence of DM in patients admitted with ACS [13], chronic kidney disease (CKD) was defined as an estimated glomerular filtration rate (eGFR) of <60 ml/min at admission [14]. DM was defined as history of elevated glucose concentrations treated by dietary control or using glucose-lowering drugs. History of congestive heart failure (CHF) was defined according to the European society of cardiology guidelines (ESC) [15].

### Endpoint definition

According the recommendation of the Academic Research Consortium regarding stent trials [16], the following endpoints were investigated: (1) cardiovascular death according to the TIMI-definition (http://www.timi.org), as well as any death that could not be attributed to non-cardiovascular reasons (2) nonfatal myocardial infarction according to the TIMI-definition (http://www.timi.org) as well as (3) any unplanned revascularization. Additionally (4) definite and probable stent thrombosis (ST) according to the academic research consortium definition [16], (5) unstable angina defined as ischemic symptoms without elevation of troponin I above the upper limit of normal and (6) transient ischemic attack (TIA) [17] or stroke, defined as cerebral infarction were recorded. Data were collected prospectively and entered into a database. Follow-up information was obtained by contacting patients after 6 months (±2 months). Source documents of all possible events were collected.

### Laboratory parameters

Blood samples for glucose, high sensitive C-reactive protein (CRP) and creatinine were obtained at hospital admission and high- and low-density lipoprotein (HDL and LDL) cholesterol and triglycerides were collected at the first day after admission in fasting condition. A certified clinical laboratory at our hospital performed the measurement of that laboratory parameter. Blood samples for GDF-15 were collected before coronary angiography. Blood samples for detection of GDF-15 were centrifuged immediately after collection and were stored at −80 °C until measurement. GDF-15 plasma concentrations were measured using a quantitative sandwich ELISA kit (Quantikine ELISA, R&D Systems) with inter- and intra-assay CVs of <6 and 2.8 %, respectively.

### Statistical analysis

Continuous data are expressed as proportions and continuous data as mean ± standard error of the mean. Categorical variables were compared by Chi square test, while analysis of variance (ANOVA) for continuous variables. Determinates of GDF-15 levels were tested using a univariate binary logistic regression analysis. All variables, which were tested significant in the univariate model, were inserted in a multivariate binary logistic regression model with inclusion method. Results of binary logistic regression analysis were presented as odds ratio (OR) and 95 % confidence interval (CI). Receiver operating characteristic (ROC) analysis was performed to determine whether GDF-15 plasma levels predict death and ischemic endpoints. All statistical tests were 2-sided and statistical significance was accepted if the p value was <0.05. All statistical analyses were performed using Software Package Social Sciences (IBM, SPSS).

## Results

The median (25–27th percentile) of GDF-15 levels was 1212.8 pg/ml (833.2–1957 pg/ml) in our study population. Patient’s demographic and clinical presentation data are depicted in Table 1. Patients with STEMI were significantly more smokers than those with SCAD and NSTEMI patients. On the other side patients with SCAD had higher cardiovascular risk profile in terms of significantly higher rates of hyperlipidemia, lower HDL-cholesterol and previous myocardial infarction (Table 1).

**Table 1:** Baseline and demographic parameters

Variable	SCAD (n = 189)	STEMI (n = 121)	NSTEMI (n = 163)	P value
Age	65.2 ± 0.786	61.9 ± 1.34	64.9 ± 1.05	0.067
Age >75 (%)	41 (21.7)	29 (24)	45 (27.6)	0.433
Female gender (%)	55 (29.1)	41 (33.9)	54 (33.1)	0.60
Hypertension (%)	170 (90)	98 (81)	139 (85.3)	0.058
Hyperlipidemia (%)	160 (85.1)	83 (68.6)	129 (79.1)	0.002
Current smoking (%)	39 (20.6)	44 (36.4)	52 (31.9)	0.006
DM (%)	58 (30.9)	28 (23.1)	50 (30.7)	0.27
Family history of CAD (%)	77 (41)	45 (37.2)	62 (38)	0.766
Previous MI (%)	63 (33.5)	19 (15.7)	42 (25.8)	0.002
CKD (%)	33 (17.5)	19 (15.7)	30 (18.4)	0.83
GDF-15 pg/ml	1421.1 ± 74.9	2189.2 ± 206.7	1695 ± 114.8	<0.0001
BMI (Kg/m^2^)	28.51 ± 0.35	28 ± 0.43	27.97 ± 0.35	0.499
e-GFR (ml/min)	65.27 ± 0.71	63.65 ± 1.04	63.57 ± 1.03	0.31
CRP (mg/dl)	7.83 ± 2.28	17.42 ± 3.88	11.05 ± 2.26	0.062
Cholesterol (mg/dl)	178.5 ± 3.66	184.25 ± 3.89	182.73 ± 4.16	0.56
HDL-cholesterol (mg/dl)	46.04 ± 1.11	41.01 ± 1.17	43.42 ± 1.13	0.009
LDL-cholesterol (mg/dl)	102.34 ± 2.88	113.58 ± 3.63	109.88 ± 3.44	0.051
Triglycerides (mg/dl)	148.73 ± 7.29	143.17 ± 10.81	163.78 ± 12.52	0.38
Systolic blood pressure (mmHg)	137.8 ± 1.5	139.4 ± 2.5	144.2 ± 2.4	0.070
Diastolic blood pressure (mmHg)	80.1 ± 0.91	81.9 ± 1.7	81.6 ± 1.3	0.507
Hospitalization days	4.84 ± 0.31	12.94 ± 1.22	7.32 ± 0.58	<0.0001

*DM* diabetes mellitus, *CAD* coronary artery disease, *CKD* chronic kidney disease, *GDF-15* growth differentiation factor 15, *BMI* body mass index, *e-GFR* estimated glomerular filtration rate, *CRP* high sensitive C-reactive protein, *HDL* high-density lipoprotein, *LDL* low-density lipoprotein

**Fig. 1:**
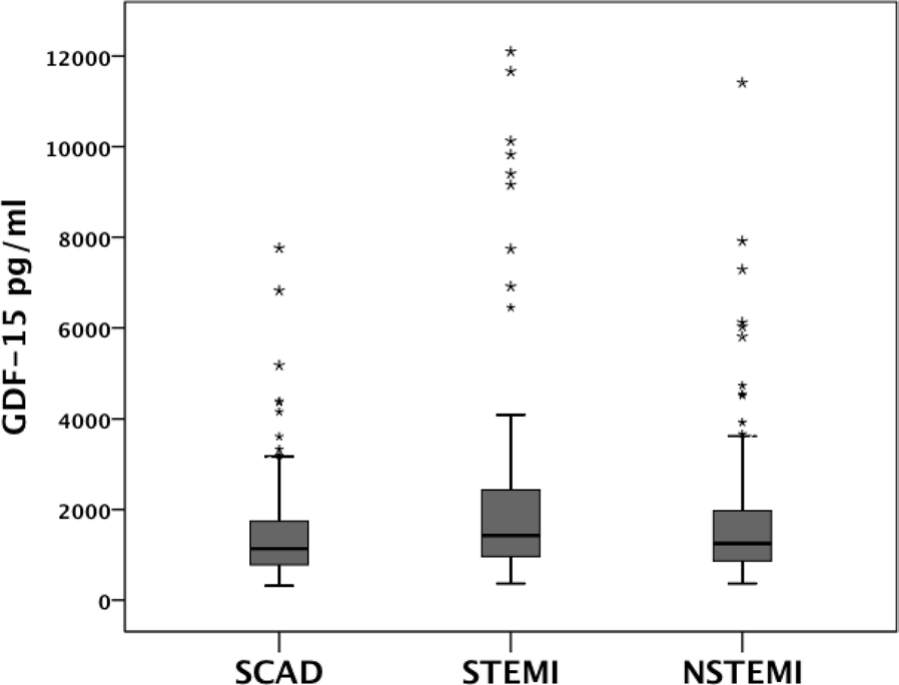
GDF-15 plasma levels in pg/ml in patients with stable coronary artery disease (SCAD), ST-elevation myocardial infarction (STEMI) and non-ST-elevation myocardial infarction (NSTEMI) (P < 0.0001 for STEMI vs. SCAD)

**Fig. 2:**
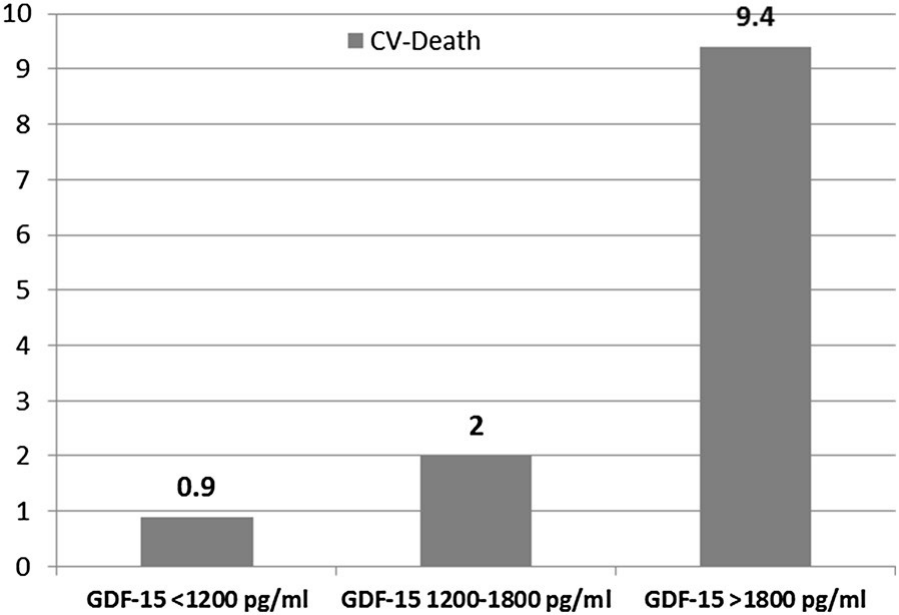
Column diagram of cardiovascular death rates among the three established thresholds for GDF-15 plasma levels [6] in patients with CAD undergoing PCI. The numbers above the columns are expressed as percent (P < 0.0001)

Additionally patients with SCAD were trend-wise older than those with ACS (STEMI and NSTEMI patients). Patients with STEMI had significantly more hospitalization days compared to those with SCAD and NSTEMI (Table 1). GDF-15 plasma levels were significantly higher in patients with STEMI compared to SCAD and NSTEMI groups (Fig. 1; Table 1). Patients with ACS (STEMI and NSTEMI together) showed significant higher proportion of GDF-15 plasma levels above 1800 pg/ml than those with SCAD (68.3 vs. 31.7 % respectively, P = 0.037). Those patients with GDF-15 plasma levels >1800 pg/ml showed higher CV-death rates compared with those patients with GDF-15 <1200 and 1200–1800 pg/ml (Fig. 2).

### Determinants of elevated GDF-15

**Fig. 3:**
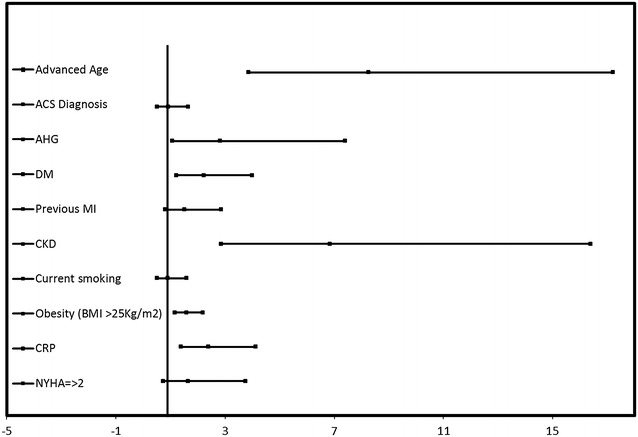
Forrest plot of multivariate logistic regression analysis of determinants of elevated GDF-15 plasma levels in patients with CAD

In univariate analysis advanced age (age >75 years), ACS on presentation, history of myocardial infarction (MI), current smoking status, elevated BMI (>25 kg/m^2^), AHG in those patients with ACS, DM, CKD, CRP and NYHA ≥2 were significant determinants of elevated GDF-15 plasma levels (Table 2). In a multivariate analysis advanced age, DM, AHG, CRP and CKD were independent predictors of elevated GDF-15 levels (Table 2; Fig. 3).

**Table 2:** Unadjusted and adjusted OR for elevated GDF-15 in patients with CAD undergoing PCI

Variable	Unadjusted OR and 95th CI	P value	Adjusted OR and 95th CI	P value
Advanced age (>75 years)	10.754 (6.003–19266)	<0.0001	8.262 (3.852–17.722)	<0.0001
Female gender	1.414 (0.958–2.087)	0.081		
ACS diagnosis	1.513 (1.045–2.191)	0.028	0.912 (0.506–1.645)	0.760
AHG in patients with ACS	3.297 (1.567–6.938)	0.002	2.815 (1.070–7.403)	0.036
DM	1.932 (1.286–2.903)	0.002	2.210 (1.222–3.995)	0.009
Previous MI	1.606 (1.060–2.432)	0.025	1.514 (0.806–2.844)	0.197
CKD	12.939 (6.071–27.574)	<0.0001	6.846 (2.858–16.400)	<0.0001
NYHA ≥2 at presentation	1.984 (1.050–3.746)	0.035	1.647 (0.726–3.739)	0.233
Current smoking	1.930 (1.284–2.901)	0.002	0.903 (0.512–1.595)	0.726
Obesity (BMI >25 kg/m^2^)	1.606 (1.050–2.455)	0.029	2.184 (1.149–4.149)	0.017
Elevated CRP at admission	1.831 (1.206–2.781)	0.005	2.380 (1.377–4.114)	0.002

*OR* odds ratio, *CI* confidence interval, *ACS* acute coronary syndrome, *AHG* acute hyperglycemia, *DM* diabetes mellitus, *MI* myocardial infarction, *CKD* chronic kidney disease, *NYHA* New York Heart Association, *BMI* body mass index, *CRP* high sensitive C-reactive protein

### ROC analysis of GDF-15 plasma levels

**Fig. 4:**
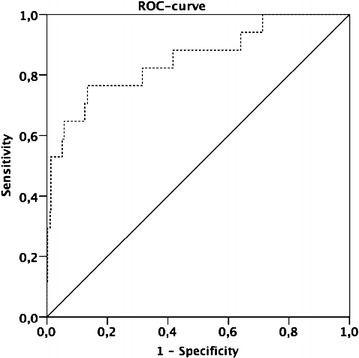
Receiver operating curve (ROC) for GDF-15 and death during a mean follow-up of 188 (177.2–243) days (area under the curve (AUC) 0.852, CI 0.745–0.960, P < 0.0001)

GDF-15 was a precise predictor of cardiovascular mortality according to the ROC Analysis [area under the curve (AUC) 0.852, CI 0.745–0.960, P < 0.0001] (Fig. 4). The best cut-off was 2094.67 pg/ml with a sensitivity of 76 % and specificity of 80 % (Fig. 4).

GDF-15 was only weakly predictive of the combined ischemic endpoint (AUC 0.473, CI 0.387–0.560, P 0.044).

## Discussion

The main results of the present study are: first, patients with ACS especially those with STEMI show the highest GDF-15 levels compared to those with SCAD. Second, several factors or conditions are independent determinants of elevated GDF-15 levels in patients with CAD who undergo PCI (advanced age, DM, AHG, inflammation and CKD).

GDF-15 is a member of the transforming growth factor ß family [1]. Under healthy conditions GDF-15 is rarely expressed [5]. In case of oxidative stress and inflammation, GDF-15 expression is up-regulated [18, 19]. Several parameters and conditions have been introduced as inducers of GDF-15 secretion [3, 4, 8, 20–22]. Patients with CAD undergoing bypass surgery are at increased risk to develop acute kidney injury when their pre-operative GDF-15 levels were elevated [23]. In patients with DM, GDF-15 predicts worsening of kidney function measured by albminuria [3]. Several investigations evidenced a relation between GDF-15 and markers of metabolic dysfunction e.g. impaired fasting glucose [8], insulin resistance and glucose metabolism [4], obesity [24], inflammation [1] and finally aging [25]. Our findings correlate with those observations as we found advanced age, DM, obesity (BMI >25 kg/m^2^), CRP and CKD were independent predictors of elevated GDF-15 in patients with CAD who needs PCI. Our investigation extends that knowledge in terms of AHG (Table 2). This is to the best of our knowledge a unique finding of the present study. AHG occurs in nearly 25 % of patients with ACS without history of DM [26, 27]. AHG has been linked to worse outcome in patients with ACS [28, 29]. AHG remotes ischemic preconditioning in experimental conditions [30]. In an in vitro study, hyperglycemia induced GDF-15 expression, which resulted in umbilical endothelial cell apoptosis [31]. Until today, the reason for elevated glucose levels in the setting of ACS is still matter of debate. Our investigation support the hypothesis that GDF-15 is linked to chronic hyperglycemia in case of DM patients and also to acute deteriorations in glucose metabolism reflected by AHG.

GDF-15 is even found in infracted myocardium as well as atherosclerotic plaque [5, 18, 19]. However, GDF-15 is not a typical marker of myocardial necrosis e.g. troponin [6, 7] and could not be used as a marker for detection of acute myocardial necrosis [6, 7]. GDF-15 has been introduced as a prognostic marker in patients with ACS especially those with NSTEMI [6, 7]. Additionally a sub-study of the ICTUS trial showed a predictive role for GDF-15 in patients with high risk NSTEMI [5]. In patients with STEMI a recent sub-study of the PLATO trial showed predictive impact of admission GDF-15 for future spontaneous MI and death [32]. A further analysis of the PLATO trial evidenced a predictive role for GDF-15 in patients with ASC undergoing, but not in those without invasive management [33]. Similarly in STEMI patients who underwent medical reperfusion therapy, GDF-15 levels were predictive of worse prognosis [2]. Wollert et al. found in a sub-investigation of the GUSTO IV trial that GDF-15 was a strong predictor of composite endpoint of death and MI [6]. However in that study the predictive power for the combined endpoint was mainly driven by the endpoint death and not MI [6]. Similar to those investigations, we found GDF-15 measured before coronary intervention in patients with CAD in stable and acute setting is a powerful predictor for death. However, GDF-15 was proven not predictive of combined ischemic endpoints of (non-fatal MI, UA, unplanned revascularization, ST, TIA and stroke). The main differences occurred in our vs. previous investigations could be explained by the fact that we investigated CAD patients in chronic as well as acute setting undergoing revascularization via PCI (PCI performed in 100 % of the study population) compared to earlier studies performed by Wollert and Kempf [2, 6] In those investigations patients were treated with thrombolytics or in less frequency received coronary intervention. Additionally the more recent studies from the PLATO investigators [32, 33] reflect more current guidelines and standard of care (in-hospital PCI and CABG ~61 and 4.5 % respectively).

*Limitations* our study has several limitations, which should be taken into account while interpreting the results. First, this is a single center investigation with a relative small sample size. Second, other than previous investigations we examined a mixed population of patients with proven CAD who needed a revascularization procedure via stent implantation. Therefore, our results should not be extrapolated to those patients treated medically, those who needed coronary artery bypass grafting as revascularization option and those with ACS treated medically only after evidencing no significant lumen narrowing of epicardial vessels in the coronary angiography. Finally, investigating the predictive value of a marker based on a single time point collection (snapshot) in a small patient population should be interpreted with caution and should be confirmed in further studies with higher sample size.

## Conclusion

In patients with CAD undergoing PCI with stent implantation, GDF-15 is determined by advanced age, acute and chronic hyperglycemia, inflammation and chronic kidney disease. GDF-15 is a valuable predictor of CD-death in a population of CAD patients after PCI.
